# Non-invasive Serological Monitoring for Crohn’s Disease Postoperative Recurrence

**DOI:** 10.1093/ecco-jcc/jjac076

**Published:** 2022-06-11

**Authors:** Amy L Hamilton, Peter De Cruz, Emily K Wright, Thierry Dervieux, Anjali Jain, Michael A Kamm

**Affiliations:** Department of Gastroenterology, St Vincent’s Hospital and Department of Medicine, University of Melbourne, Melbourne, VIC, Australia; Department of Gastroenterology, St Vincent’s Hospital, Department of Gastroenterology, Austin Health and Department of Medicine, University of Melbourne, Melbourne, VIC, Australia; Department of Gastroenterology, St Vincent’s Hospital and Department of Medicine, University of Melbourne, Melbourne, VIC, Australia; Prometheus Laboratories, San Diego, CA, USA; Prometheus Laboratories, San Diego, CA, USA; Department of Gastroenterology, St Vincent’s Hospital and Department of Medicine, University of Melbourne, Melbourne, VIC, Australia

**Keywords:** Crohn’s disease, serology, mucosal healing, postoperative recurrence, disease monitoring

## Abstract

**Introduction:**

Crohn’s disease recurs after intestinal resection. This study evaluated accuracy of a new blood test, the Endoscopic Healing Index [EHI], in monitoring for disease recurrence.

**Methods:**

Patients enrolled in the prospective POCER study [NCT00989560] underwent a postoperative colonoscopic assessment at 6 [2/3 of patients] and 18 months [all patients] following bowel resection, using the Rutgeerts score [recurrence ≥i2]. Serum was assessed at multiple time points for markers of endoscopic healing using the EHI, and paired with the Rutgeerts endoscopic score as the reference standard.

**Results:**

A total of 131 patients provided 437 serum samples, which were paired with endoscopic assessments available in 94 patients [30 with recurrence] at 6 months and 107 patients [44 with recurrence] at 18 months. The median EHI at 6 months was significantly lower in patients in remission [Rutgeerts <i2] than those with recurrence; *p* = 0.033. The area under the receiver operating curve [AUROC] for EHI to detect recurrence at 6 months was comparable to that of faecal calprotectin [0.712 vs 0.779, *p* = 0.414]. EHI of <20 at 6 months had a negative predictive value of 75.7% (95% confidence interval [CI] 58.8–88.2), and sensitivity of 70% [95% CI 50.6–85.3] for detecting recurrence. Combining all time points, an EHI <20 had a negative predictive value of 70.3%. Changes in EHI significantly associated with changes in Rutgeerts scores over the 18 months.

**Conclusions:**

The non-invasive multi-marker EHI has sufficient accuracy to be used to monitor for postoperative Crohn’s disease recurrence. A monitoring strategy that combines EHI with ileocolonoscopy, with or without faecal calprotectin, should now be prospectively tested.

## 1. Introduction

Within 1 year of an intestinal resection to remove all macroscopic Crohn’s disease [CD], endoscopically identifiable disease recurrence occurs in up to 90% of patients,^1–3^ and a further operation is required in up to 70% of patients within 10 years of their initial surgery.^4^ The severity of endoscopic recurrence at the anastomosis 1 year after surgery is predictive of later clinical recurrence and the need for surgery.^5^ Endoscopic assessment using ileocolonoscopy remains the gold standard for detection of recurrence, but is expensive, invasive, cannot be repeated frequently, and is not agreeable to many patients.^6^

In the Post Operative Crohn’s Endoscopic Recurrence [POCER] study, endoscopic monitoring and treatment of early disease recurrence reduced Crohn’s disease progression.^7^ Endoscopically identified disease recurrence and progression occur before clinical symptoms develop.^7^ Measurement of serum C-reactive protein [CRP] is insensitive for disease recurrence,^8^ partly because of the low disease burden with early recurrence and partly because some patients with active Crohn’s disease do not develop an elevated C-reactive protein with active disease. Magnetic resonance enterography [MRE] is sensitive for diagnosing severe [Rutgeerts i3-i4] recurrence, but is less sensitive for the identification of early recurrent lesions.^8,9^ Intestinal ultrasound has moderate sensitivity for detecting disease recurrence^10^ but is dependent on visualisation of the anastomosis, body habitus of the patient, and the experience of the operator. Faecal calprotectin [FC] is currently the best studied biomarker of recurrence, and has good sensitivity and negative predictive value for detecting postoperative recurrence.^11–14^ Many patients dislike stool-based tests and would preferentially undergo a blood test instead of providing a faecal sample.^6,15^

An accurate, simple, serum, non-invasive biomarker sensitive for disease recurrence would assist in the monitoring for disease recurrence after surgery and would improve patient compliance. Compliance with recommended postoperative endoscopic surveillance intervals is generally poor, with only 30–54% of patients undergoing a colonoscopy within 12 months of surgery.^16–19^

The Endoscopic Healing Index [EHI; Monitr™ Panel] is a sensitive measure of intestinal mucosal damage and repair, validated against endoscopy, that combines 13 biomarkers of matrix remodelling, angiogenesis, cell adhesion, immune recruitment, and growth factors such as TGF-α.^20^ The markers are combined with variable weightings to produce an endoscopic healing index [EHI] score that ranges from 0 to 100.

EHI has been previously validated in a prospective cohort of patients being treated for active Crohn’s disease in the TAILORIX clinical trial, as well as a separate cohort from a tertiary clinical practice.^21,22^ An index value of less than 20 was found to exclude the presence of endoscopic inflammation with a sensitivity of 83.2% to 97.1%.^20^ A value of 50 or greater had a specificity of 87.8% to 100% for identifying the presence of endoscopic inflammation with a Simple Endoscopic Score for Crohn’s Disease [SES-CD] ≥3.^20^ Specificity of indices between 20 to 50 progressively increased as the values approached 50, indicating a higher probability of endoscopic inflammation with increasing EHI values.

The active disease setting, in which the goal is to detect mucosal healing in response to treatment, differs from the postoperative setting in which the goal is to detect the early recurrence of mucosal inflammation. This study assesses the utility of the Endoscopic Healing Index for the identification of early endoscopic recurrence in a well-characterised prospective cohort of Crohn’s disease patients.

## 2. Materials and Methods

### 2.1. Clinical study

The POCER Study has been reported previously.^7^ In this prospective study of 174 patients undergoing resection of all macroscopic Crohn’s disease, 131 patients had at least one endoscopic assessment paired with a serum sample, and form the basis of this analysis. All patients underwent resection of all macroscopic luminal Crohn’s disease [Table 1] and had an endoscopically assessable primary anastomosis.^7^ Patients were randomised [2:1 ratio] to a colonoscopy and endoscopic assessment at 6 months [active care] or to best standard drug therapy and no colonoscopy at 6 months. All patients underwent a colonoscopic assessment at 18 months postoperatively [Supplementary Figure 1].

**Table 1.** Patient demographics at baseline.

	*n *= 131 [437 Samples]	
	*n*	%
Male	61	46.6
Age > 40 years	55	42.0
Age, median [years]:	36.2	
Interquartile range [IQR]	26.0–46.5	
Age at diagnosis [years]		
≤16 years	14	10.7
17–40 years	99	75.6
>40 years	18	13.7
Duration of Crohn’s disease, median [years]	9	
Interquartile range [IQR]	3–16	
≥10 years	64	48.9
Disease location at surgery		
Ileum only [L1]	71	54.2
Colon only [L2]	10	7.6
Ileum and colon [L3]	50	38.2
Disease phenotype at surgery		
B1 [inflammatory]	13	9.9
B2 [stricturing]	43	32.8
B3 [penetrating]	75	57.3
Indication for surgery		
Failure of drug therapy	28	21.4
Obstruction	34	26.0
Perforation	69	52.7
Number of prior surgical resections		
0	95	72.5
1	24	18.3
2	6	4.6
3 or more	6	4.6
Resection type		
Ileocaecal resection	91	69.5
Isolated ileal resection	7	5.3
Ileocaecal and proximal ileal resection	13	9.9
Colectomy	3	2.3
Stoma closure	15	11.5
Stoma closure + ileocaecal or ileal resection	2	1.5
Smoking status		
Active smoker	40	30.5
Past smoker	35	26.7
Never smoker	56	42.7
Immediate postoperative baseline drug therapy		
Metronidazole alone	23	17.6
Thiopurine	76	58.0
Adalimumab	32	24.4
Baseline CDAI	118	
CDAI >150	91	69.5
CDAI >200	76	58.0
6-month endoscopic scores	94	
Median SES-CD [IQR]	3 [4]	
Range [minimum—maximum]	0–15	
18-month endoscopic scores	107	
Median SES-CD [IQR]	3 [1]	
Range [minimum—maximum]	0–17	

CDAI, Crohn’s Disease Activity Index.

Patients were classified according to the Montreal Classification.^23^ Patients received drug therapy according to stratification based on pre-operative risk of recurrence and their study arm allocation.^7^ Patients were deemed ‘high risk’ at POCER study entry if they had ≥1 of three risk factors: current smoking, penetrating disease phenotype, or previous surgical resection. Patients were deemed ‘low risk’ if they had none of these three risk factors for early disease recurrence.^7^

For assessment of the endoscopic healing index, 437 serum samples were prospectively obtained: 118 at peri-operative baseline [prior to or within 6 weeks of surgery], 124 at 6 months, 88 at 12 months, and 107 at 18 months postoperatively; 131 patients (108 [82.4%] high risk) provided one or more serum samples and had at least one endoscopic assessment. A total of 77 patients provided all four serum samples. Baseline characteristics of the cohort are shown in Table 1.

### 2.2. Endoscopic assessments and changes in treatment

Endoscopic assessment was undertaken using the Rutgeerts score,^5^ with recurrence defined as a score ≥i2, performed by experienced assessors and confirmed by agreement of two blinded central readers [MAK and PDC].

The Rutgeerts score assesses both the surgical anastomosis and the proximal ileum and defines postoperative disease as: i0 [no ulcers]; i1 [≤5 aphthous lesions]; i2 [>5 aphthous lesions or large lesions at anastomosis; i3 [diffuse ileitis]; or i4 [large ulcers with diffuse inflammation and larger lesions, and/or anastomotic narrowing].^5^

Patients in the active care arm with endoscopic recurrence at 6 months intensified medical therapy: low-risk patients commenced a thiopurine, and high-risk patients on a thiopurine from baseline commenced combination therapy with the addition of adalimumab 40 mg fortnightly. Patients on adalimumab monotherapy increased dosing to 40 mg weekly.^7^

### 2.3. Sample collection and testing

Serum testing was performed using a reference clinical laboratory accredited by the College of American Pathologists and immunoassay determinations [Monitr™ Panel, Prometheus Laboratories, San Diego, CA], blinded to patient randomisation or characteristics. The panel measures the following biomarkers: CEACAM1, VCAM1, CRP, SAA1, Ang-1, Ang-2, MMP-1, -2, -3, -9, EMMPRIN, TGF-α, IL-7 [Supplementary Table 1]. The EHI was developed using a training dataset [335 samples from 278 patients] with 47 markers initially selected and analysed against the Crohn’s Disease Endoscopic Index of Severity [CDEIS]^24^ and the Simple Endoscopic Score for Crohn’s Disease [SES-CD].^25^ The logistic regression model developed [comprising the 13 best-performing markers] was then validated with data from the TAILORIX trial and a separate prospective cohort of CD patients.^26^

Faecal calprotectin was measured using a quantitative enzyme immunoassay [fCAL™, Bühlmann, Schonenbuch, Switzerland], expressed as micrograms per gram of stool.

### 2.4. Endpoints

The primary analysis assessed the overall accuracy of the EHI score at various cut-offs for the presence or absence of endoscopic recurrence [Rutgeerts ≥i2] and for distinguishing between complete macroscopic mucosal normality [Rutgeerts i0] and severe recurrence [Rutgeerts i3 or i4].

Secondary outcomes of interest included other endoscopic scores, faecal calprotectin, and C-reactive protein [CRP]. The faecal calprotectin was defined as elevated or within the normal range based on our previous work in the postoperative setting, which established a sensitivity of 89% and specificity of 58% of calprotectin concentration 100 µg/g for recurrence of Rutgeerts i2 or greater.^12^

### 2.5. Statistical analysis

Results are reported according to the Standards for Reporting of Diagnostic Accuracy Studies [STARD] statement.^27^ Data were analysed using STATA Version 15 [StataCorp, TX, USA] and R version 3.6.2.^28^ Univariate analysis of association with patient characteristics was performed with the chi-square or Fisher’s exact test; continuous variables were assessed using the Wilcoxon rank sum test, the Wilcoxon signed rank test for serial measurements, or the Kruskal–Wallis test if more than two groups were compared. A linear mixed effect model [using R packages nlme^29^ and MuMIn^30]^ was used to assess the longitudinal changes in Rutgeerts score relative to EHI, with EHI and study arm as fixed effect and the patient as random effect. The EHI was transformed into a coded variable with groups EHI <20 [value of 0], 20–50 [value of 1], and >50 [value of 2] for modelling the relationship between stepwise increase in EHI categories and changes in the Rutgeerts score. The estimation method used for the modelling was restricted maximum likelihood [REML].

Test performance was assessed by calculating positive predictive values [PPV] and negative predictive values [NPV] for prediction of endoscopic recurrence. Receiver operator characteristic [ROC] analysis was performed using R packages pROC^31^ and epiR^32^ [sensitivity v 1-specificity], and the area under the receiver operating characteristic curve [AUROC] calculated to assess the performance of the EHI in identifying patients with endoscopic recurrence. Comparisons of the ROC curves obtained from EHI, CRP, and calprotectin were performed using the Delong method.^33^ EHI assessments were performed in three cohorts, all with similar baseline demographics [Tables 1 and 2].

**Table 2.** Remission and recurrence rates across the three analysis cohorts.

Cohort	Patients, *n*	Samples, *n*	Patients with endoscopic recurrence at 6 months	Patients with endoscopic recurrence at 18 months
Cross-sectional	131	437	30 [31.9%]	44 [41.1%]
Test comparison cohort	114	275	22 [34.4%]	24 [35.8%]
Test comparison cohort limited to endoscopic assessment at 6/18 months	94	131	22 [34.4%]	24 [35.8%]
Longitudinal [active care arm only]	70	264	25 [35.7%]	23 [32.9%]

The *cross-sectional cohort* [all samples, all patients] involved calculation of median EHI at all time points and in relation to baseline patient characteristics. Serum samples paired with an endoscopic assessment at 6 or 18 months were considered separately and together [Supplementary Figures 1 and 2].

The *test comparison cohort* included all samples that had simultaneous EHI, CRP, and calprotectin measurements at any time point.

The *longitudinal cohort* consisted of patients in the active care POCER study arm with EHI paired with a colonoscopy performed at both 6 and 18 months. The dynamics of EHI over time was assessed in patients who had a colonoscopy at both 6 and 18 months, to assess association with disease progression in those patients who did and those who did not increase their treatment intensity.

### 2.6. Ethical considerations

The POCER Study [including this sub-analysis] was approved by the Human Research Ethics Committee of St Vincent’s Hospital, Melbourne [HREC-A 077/09] and was registered with ClinicalTrials.gov [NCT00989560]. All patients provided written informed consent.

## 3. Results

### 3.1. Demographics and baseline characteristics

Baseline patient demographics for the 131 patients are shown in Table 1.

Of the baseline samples [*n* = 118], 64 [54.3%] were obtained immediately preoperatively and 54 [45.7%] in the 6 weeks following surgery (median days after surgery, 9; interquartile range [IQR] 5–17 days]. Endoscopic recurrence [Rutgeerts ≥i2] occurred in 31.9% [30/94] of patients in the active arm at 6 months and in 41.1% [44/107] of all patients at 18 months [Table 2].

### 3.2. Baseline Endoscopic Healing Index in relation to Rutgeerts score

The change in Endoscopic Healing Index [EHI] between postoperative baseline [*n* = 37] and 6 months did not differ between those with [*n *= 9] and without [*n* = 28] endoscopic recurrence: -19 [IQR -35 to -2] vs -12 [IQR -21.5 to -4]; *p* = 0.491. Similarly, the change in EHI between postoperative baseline [*n *= 47] and 18 months did not differ between those with [*n* = 16] and without [*n* = 31] endoscopic recurrence: -12 [IQR -21 to -4] vs -20 [IQR -31 to -5]; *p* = 0.226.

### 3.3. Cross-sectional EHI in relation to Rutgeerts score

**Figure 1.**
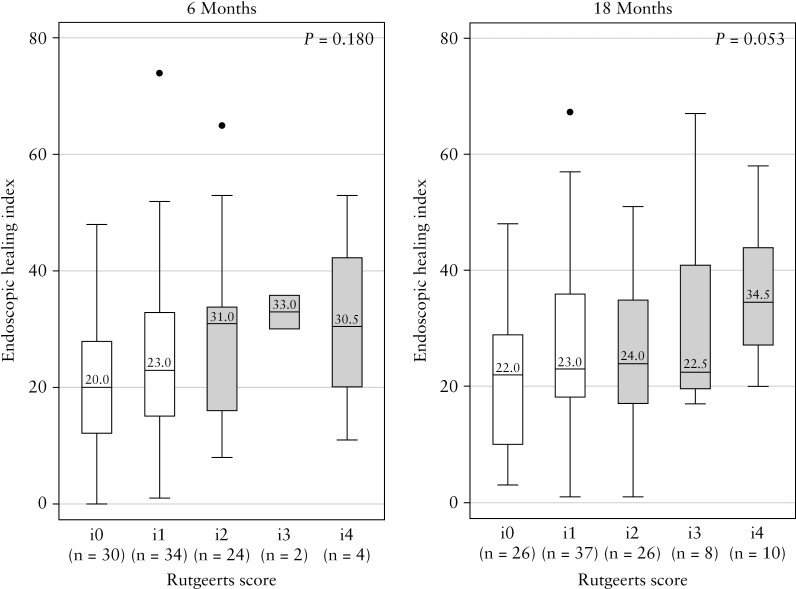
Endoscopic Healing Index by Rutgeerts score at 6 and 18 months in the cross-sectional cohort. Remission shown by white bars, recurrence by shaded bars; *p-v*alues for Kruskal–Wallis test.

The median EHI tended to increase with increasing Rutgeerts score at both 6- and 18-month endoscopies [cross-sectional cohort; Figure 1].

**Figure 2.**
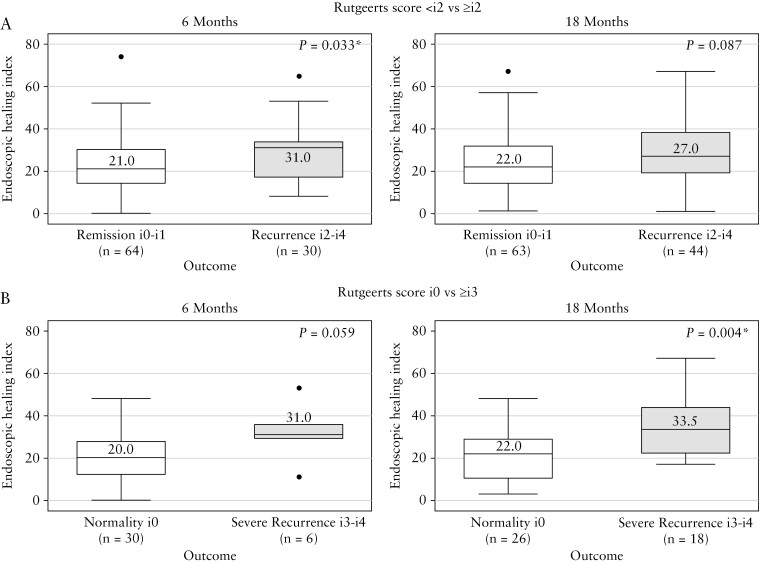
Endoscopic Healing Index values [cross-sectional cohort] for endoscopic remission vs recurrence and mucosal normality vs severe recurrence at both 6 [Panel A] and 18 months [Panel B]; *p-v*alues shown for Wilcoxon rank sum test.

At 6 months, median EHI was significantly lower in patients in remission when compared with patients with recurrence (i0 + i1 vs i2 + i3 + i4, that is <i2 vs ≥i2, 21 [IQR 14–30.5] vs 31 [IQR 17–34]; *p* = 0.033; Figure 2A left panel).

At 6 months the EHI was lower in those with mucosal normality than in those with severe recurrence (i0 vs i3 + i4, EHI 20 [IQR 12–28] vs 31 [IQR 29–36]; *p* = 0.059; Figure 2B left panel). The EHI was lower in those in remission [i0 plus i1] compared with those with severe disease [i3 plus i4] at 6 months: 21 [IQR 14–30.5] vs 31 [IQR 29–36]; *p* = 0.086. Patients with mucosal normality [Rutgeerts i0] had a lower EHI than patients with any macroscopic disease [Rutgeerts i1–i4] (i0 vs ≥i1: EHI 20 [IQR 12–28] vs 27 [IQR 16–24]; *p* = 0.055).

At 18 months, the median EHI was lower in those in remission compared with those with recurrence (<i2 vs ≥i2: EHI 22 [IQR 14–32] vs 27 [IQR 19–38.5]; *p* = 0.087; Figure 2A right panel) and significantly lower in patients with mucosal normality than those with severe recurrence (i0 vs i3 + i4, EHI 22 [IQR 10–29] vs 33.5 [IQR 22–44]; *p* = 0.004; Figure 2B right panel). The EHI was also significantly lower at 18 months in those with remission [i0 plus i1] compared with severe recurrence [i3 plus i4] at 18 months: 22 [IQR 14–32] vs 33.5 [IQR 22–44]; *p* = 0.013. At 18 months, patients with mucosal normality had a significantly lower EHI compared with those who had any macroscopic disease [Rutgeerts i1-i4] (i0 vs ≥i1: EHI 22 [IQR 10–29] vs 24 [IQR 18–38]; *p* = 0.045).

When the 6- and 18-month measurements were combined, the median EHI was lower in remission versus those with recurrence (<i2 vs ≥i2, EHI 22 [IQR 14–31] vs 29.5 [IQR 18–36]; *p* = 0.005), as well as in those with remission [i0 plus i1] compared with severe recurrence [i3 plus i4]: 22 [IQR 14–31] vs 32.5 [IQR 22.5–41.5]; *p* = 0.001.

### 3.4. EHI test performance

Linear mixed modelling assessed the association between the longitudinal changes in Rutgeerts score [6- and 18-month endoscopies, 131 patients, 201 samples] and categorical EHI values [<20; 20–50; >50], while controlling for study arm [active/standard care]. EHI associated significantly with Rutgeerts score. For each longitudinal increase in EHI category there was a 0.48 ± 0.14 [R^2^ = 0.11, *p* = <0.001; AIC = 607.6] point increase in Rutgeerts score.

**Figure 3.**
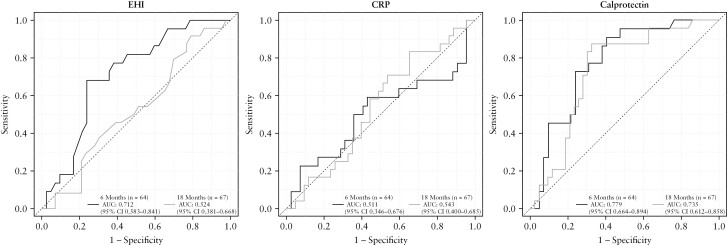
AUROC curves for endoscopic remission versus recurrence [Rutgeerts <i2 vs ≥i2] for Endoscopic Healing Index, C-reactive protein and calprotectin at 6 and 18 months [calprotectin cohort]. AUROC, area under the receiver operating curve.

Sensitivity, specificity, positive predictive value [PPV], and negative predictive values [NPV], false-negative and false-positive rates of the EHI for detection of endoscopic recurrence [Rutgeerts score ≥i2] at 6 months, 18 months, and combined 6 and 18 months are shown in Table 3. The ‘Youden’ selected value, a test cut-off that provides equal weighting to sensitivity and specificity for the 6- and 18-month EHI measurements combined, was 30.^34^ Areas under the curve [AUROC] are shown in Figure 3. The AUROC for EHI to identify endoscopic recurrence [<i2 vs ≥i2] in any endoscopically matched sample [at 6 and 18 months combined] was 0.62. The AUROC to discriminate between mild disease [Rutgeerts ≤i1] and severe recurrence [Rutgeerts ≥i3] was 0.71. Considering the most important clinical parameters of detecting a sufficient proportion of patients with true recurrence [sensitivity] and confidence that a negative test is correct [negative predictive value], a threshold of <20 [sensitivity of 70.3%, NPV of 70.3% for 6 and 18 months combined] was determined as the most clinically useful and appropriate to identify patients in endoscopic remission. A threshold of <10 [sensitivity of 94.6%, NPV of 81.8%] had the highest negative predictive value and could be used when a greater degree of confidence is required that there is no recurrent disease, such as when ileocolonoscopy or faecal calprotectin cannot be performed. Further longitudinal analysis of the EHI in patients within the active care arm [Supplementary Table 2, Supplementary Figure 3A], including predictive value and response to treatment step-up [Supplementary Figure 3B] are presented in the [Supplementary Material].

**Table 3.** Sensitivity, specificity, PPV, NPV [95% CI] and false positive and negative rates [%] of the EHI at different thresholds for 6 and 18 months postoperatively [Cross-sectional cohort] for endoscopic remission [<i2] vs endoscopic recurrence [≥i2]. Italics represent the Youden selected threshold values.

	EHI threshold	*N* [%] patients less than EHI threshold	*N* [%] patients in remission [<i2] with EHI less than threshold	Sensitivity	Specificity	Positive predictive value [PPV]	Negative predictive value [NPV]	False-positive rate	False-negative rate	Positive likelihood ratio [PLR]	Negative likelihood ratio [NLR]
	**6-months [*n *= 94, prevalence 31.9%]**										
Low	<10	9 [9.6]	8 [88.9]	96.7 [82.8–99.9]	12.5 [5.6–23.2]	34.1 [24.2–45.2]	88.9 [51.8–99.7]	87.5	3.3	1.105 [0.986–1.238]	0.267 [0.986–1.238]
Mid	<20	37 [39.4]	28 [75.7]	70 [50.6–85.3]	43.8 [31.4–56.7]	36.8 [24.4–50.7]	75.7 [58.8–88.2]	56.2	30	1.244 [0.905–1.712]	0.686 [0.371–1.266]
*Youden selected threshold*	*<29*	*57 [60.6]*	*45 [79.0]*	*60.0* *[40.6*–*77.3]*	*70.3* *[57.6*–*81.1]*	*48.6* *[31.9–65.6]*	*78.9* *[66.1–88.6]*	*29.7*	*40*	*2.021* *[1.254–3.256]*	*0.569* *[0.357–0.907]*
High	<50	89 [94.6]	62 [69.6]	10.0 [2.1–26.5]	96.9 [89.2–99.6]	60.0 [14.7–94.7]	69.7 [59.0–79.0]	3.1	90	3.200 [0.564–18.156]	0.929 [0.818–1.055]
	**18-months [*n* = 107, prevalence 41.1%]**										
Low	<10	13 [12.1]	10 [76.9]	93.2 [81.3–98.6]	15.9 [7.9–27.3]	43.6 [33.4–54.2]	76.9 [46.2–95.0]	84.1	6.8	1.108 [0.969–1.266]	0.430 [0.125–1.472]
Mid	<20	37 [34.6]	24 [64.9]	70.5 [54.8–83.2]	38.1 [26.1–51.2]	44.3 [32.4–56.7]	64.9 [47.5–79.8]	61.9	29.5	1.138 [0.867–1.494]	0.776 [0.446–1.350]
*Youden selected threshold*	*<34*	*79 [73.8]*	*51 [64.6]*	*36.4* *[22.4*–*52.2]*	*81.0* *[69.1*–*89.8]*	*57.1* *[37.2*–*75.5]*	*64.6* *[53.0*–*75.0]*	*19.0*	*63.6*	*1.909* *[1.005–3.627]*	*0.786* *[0.610–1.013]*
High	<50	101 [94.4]	60 [59.4]	6.8 [1.4–18.7]	95.2 [86.7–99.0]	50.0 [11.8–88.2]	59.4 [49.2–69.1]	4.8	93.2	1.432 [0.303–6.768]	0.978 [0.888–1.078]
	**6- & 18-month combined [*n *= 201, prevalence 36.8%]**										
Low	<10	22 [10.9]	18 [81.8]	94.6 [86.7–98.5]	14.2 [8.6–21.5]	39.1 [37.0–41.3]	81.8 [61.3–92.8]	85.8	5.4	1.10 [1.01–1.21]	0.38 [0.13–1.08]
Mid	<20	74 [36.8]	52 [70.3]	70.3 [58.5–80.3]	40.9 [32.3–50.0]	40.9 [36.0–46.0]	70.3 [61.1–78.0]	59.1	29.7	1.19 [0.97–1.46]	0.73 [0.48–1.09]
*Youden selected threshold*	*<30*	*128 [63.7]*	*91 [71.1]*	*50.0* *[38.1*–*61.9]*	*71.7* *[63.0*–*79.3]*	*50.7* *[41.8–59.5]*	*71.1* *[65.6–76.0]*	*28.3*	*50*	*1.76* *[1.23–2.52]*	*0.70* *[0.54–0.90]*
High	<50	190 [94.5]	122 [64.2]	8.1 [3.0–16.8]	96.1 [91.1–98.7]	54.6 [27.5–79.2]	64.2 [62.4–65.9]	3.9	91.9	2.06 [0.65–6.52]	0.96 [0.89–1.03]

EHI, Endoscopic Healing Index.

### 3.5. Comparison of EHI, calprotectin, and CRP for identification of endoscopic recurrence [Rutgeerts ≥i2]

EHI, CRP, and faecal calprotectin performances were compared with Rutgeerts score in 94 patients where both serum and stool samples were available within 45 days of endoscopy [‘test comparison cohort’].

The linear mixed model assessing the association between the longitudinal changes in Rutgeerts score [6- and 18-month endoscopies, patients = 94, 131 samples] versus categorical EHI values [<20; 20–50 and >50], faecal calprotectin [log normal], or CRP while controlling for study arm [active/standard care] revealed a significant impact of EHI and faecal calprotectin, but not of CRP, on Rutgeerts score. A longitudinal increase in EHI categories or faecal calprotectin concentrations associated with a significant increase in Rutgeerts score [estimate = 0.378 ± 0.16 and 0.259 ± 0.060, respectively, R^2^ = 0.090 and 0.169, respectively] [*p* <0.025]. In contrast, there was no significant association of CRP with Rutgeerts scores [0.086 ± 0.085, R^2^ = 0.059; *p* = 0.322].

Comparisons were also made separately at 6 and 18 months. Faecal calprotectin differed significantly between patients in endoscopic remission [Rutgeerts i0 or i1] and those with endoscopic recurrence [Rutgeerts ≥i2] at 6 months: 85.5 µg/g [IQR 36.0–193.0] vs 263.5 µg/g [IQR 163.0–540.0]; *p* = 0.0003; and at 18 months: 72.0 µg/g [IQR 39.0–326.0] vs 331.0 µg/g [IQR 159.0–550.0]; *p* = 0.002.

CRP did not differ significantly between those with endoscopic disease remission and recurrence at 6 months: <i2 vs ≥i2: 2.0 mg/L [IQR 0.6–3.6] vs 2.3 mg/L [IQR 0.4–4.5], *p* = 0.894; or at 18 months: 0.9 mg/L [IQR 0.6–2.6] vs 1.1 mg/L [IQR 1.1–2.2], *p* = 0.572.

At 6 months, the EHI [at a threshold of <20] and faecal calprotectin [at a threshold <100] had similar, albeit slightly lower, sensitivity [81.8% and 90.9%, respectively], specificity, and NPV [84.0% and 91.7%, respectively] for the detection of Rutgeerts i2 or greater disease recurrence [Table 4].

**Table 4.** Comparison of test performance in the test comparison cohort for the Endoscopic Healing Index, C-reactive protein, faecal calprotectin, and combined EHI and faecal calprotectin tests in relation to endoscopic recurrence [Rutgeerts score i2-i4] and remission [Rutgeerts score i0-i1]. All values expressed as percentages (excluding PLR and NLR); *p-*values calculated using DeLong’s test. Inter quartile ranges for each value are shown in italics.

		Cut-off	Sensitivity	Specificity	PPV	NPV	PLR	NLR	AUROC	*p-v*alue AUROC EHI vs CRP	*p-v*alue AUROC EHI vs Calprotectin
6 months	CRP	<5 mg/L	22.7	90.5	55.6	69.1	2.39	0.85	0.511	0.052	
	*IQR*		*[7.8–45.4]*	*[77.4–97.3]*	*[21.2–86.3]*	*[55.2–80.9]*	*[0.71–8.00]*	*[0.67–1.09]*			
	EHI	<20	81.8	50	46.2	84	1.64	0.36	0.712		0.414
	*IQR*		*[59.7–94.8]*	*[34.2–65.8]*	*[30.1–62.8]*	*[63.9–95.5]*	*[1.14–2.35]*	*[0.14–0.93]*			
	Calprotectin	<100 µg/g	90.9	52.4	50	91.7	1.91	0.17	0.779		
	*IQR*		*[70.8–98.9]*	*[36.4–68.0]*	*[33.8–66.2]*	*[73.0–99.0]*	*[1.35–2.69]*	*[0.05–0.67]*			
6 months combined EHI and Calprotectin	EHI	<20	72.7	64.3	51.6	81.8	2.04	0.42	0.685		
	Calprotectin	<100 µg/g									
	*IQR*		*[49.8–89.3]*	*[48.0–78.5]*	*[39.8–63.3]*	*[68.7–90.2]*	*[1.26–3.29]*	*[0.21–0.87]*			
	EHI	<10	90.9	57.1	52.6	92.3	2.12	0.16	0.740		
	Calprotectin	<100 µg/g									
	*IQR*		*[70.8–98.9]*	*[41.0–72.3]*	*[43.3–61.8]*	*[75.7–97.9]*	*[1.46–3.08]*	*[0.04–0.61]*			
18 months	CRP	<5 mg/L	16.7	86	40	64.9	1.194	0.968	0.543	0.853	
	*IQR*		*[4.7–37.4]*	*[72.1–94.7]*	*[12.2–73.8]*	*[51.1–77.1]*	*[0.374–3.819]*	*[0.781–1.202]*			
	EHI	<20	66.7	32.6	35.6	63.6	0.989	1.024	0.524		0.027
	*IQR*		*[44.7–84.4]*	*[19.1–48.5]*	*[21.9–51.2]*	*[40.7–82.8]*	*[0.696–1.404]*	*[0.503–2.084]*			
	Calprotectin	<100 µg/g	87.5	60.5	55.3	89.7	2.213	0.207	0.735		
	*IQR*		*[67.6–97.3]*	*[44.4–75.0]*	*[38.3–71.4]*	*[72.6–97.8]*	*[1.485–3.300]*	*[0.070–0.612]*			
18 months combined EHI and calprotectin	EHI	<20	62.5	74.4	57.7	78.1	2.44	0.5	0.685		
	Calprotectin	<100 µg/g									
	*IQR*		*[40.6–81.2]*	*[58.8–86.5]*	*[42.9–71.2]*	*[67.3–86.0]*	*[1.35–4.44]*	*[0.29–0.87]*			
	EHI	<10	83.3	62.8	55.6	87.1	2.24	0.27	0.731		
	Calprotectin	<100 µg/g									
	*IQR*		*[62.6–95.3]*	*[46.7–77.0]*	*[44.9–65.7]*	*[72.8–94.4]*	*[1.46–3.43]*	*[0.11–0.67]*			

EHI, Endoscopic Healing Index; PPV, positive predictive value; NPV, negative predictive value; AUROC, area under the receiver operating curve; CRP, C-reactive protein; IQR, interquartile range; PLR, positive likelihood ratio; NLR, negative likelihood ratio.

Calprotectin and EHI were combined using a threshold of calprotectin [<100 µg/g] and EHI thresholds of <10 and <20 [Table 4] at both 6 and 18 months for endoscopic recurrence [Rutgeerts ≥i2]. At 6 months, the combination of calprotectin [<100 µg/g] and EHI <20 had a higher specificity [64.3%] that either test alone [52.4% and 50.0%, respectively], but did not improve the sensitivity or NPV. However, calprotectin [<100 µg/g] and EHI <10 combined had a sensitivity of 90.9% and a high NPV of 92.3%. At 18 months, calprotectin [<100 µg/g] and EHI <10 combined had a sensitivity of 83.3% and an NPV of 87.1%.

The AUROC for EHI to differentiate i0 plus i1 [remission] versus i2 to i4 [recurrence] was 0.712 [95% CI 0.583–0.841] at 6 months and 0.524 [95% CI 0.381–0.668] at 18 months.

The AUROC for calprotectin at 6 months was 0.779 [95% CI 0.664–0.894] and at 18 months 0.735 [95% CI 0.612–0.858]. For CRP, the AUROC was 0.511 [95% CI 0.346–0.676] and 0.543 [95% CI 0.400–0.685], respectively.

When the AUROCs for EHI, calprotectin, and CRP were compared at 6 months, the AUROCs for EHI and calprotectin were both superior to CRP [*p* = 0.05, Table 4]. The AUROC for faecal calprotectin did not differ statistically when compared with EHI [0.78 vs 0.71; *p* = 0.414]. However at 18 months, the AUROC was significantly greater for calprotectin compared with EHI [*p* = 0.027]. AUROC curves for both 6 and 18 months for EHI, CRP, and calprotectin are shown in Figure 3.

## 4. Discussion

It is now accepted that proactive surveillance for postoperative recurrence is essential, and should commence within 6 months of resection. However, there are few non-invasive options with the sensitivity to identify minimal, localised, yet clinically significant postoperative disease.

This prospective longitudinal study has demonstrated that a multi-marker panel focused on gastrointestinal mucosal healing performs well in detecting or excluding recurrent disease.

Clinical indices in the postoperative setting, such as the Crohn’s Disease Activity Index, are of little value as they do not correlate with the presence of recurrent endoscopic disease.^35^ The use of blood-based biomarkers such as CRP to diagnose postoperative Crohn’s disease has been disappointing as they lack sensitivity to diagnose localised disease, but patients prefer venepuncture over other tests.^6,15,36^ Calprotectin has good sensitivity and specificity for disease activity but there are barriers to compliance, with up to 80% of patients preferring a blood test.^15^ There are also a range of proposed thresholds for calprotectin in the postoperative setting. In our prospective large cohort, a threshold calprotectin concentration of 100 ug/g was optimal, with a sensitivity of 89%. A more recent meta-analysis reported an optimal threshold of 150 ug/g with a sensitivity of 81%.^12,37^

Whereas ileocolonoscopy remains the gold standard for assessment of endoscopic disease activity, it cannot assess more proximal disease, is not always practical, cannot be performed frequently, and has the poorest acceptability to patients.^6^

The EHI is still elevated immediately postoperatively. This most likely relates to the effect of surgery on this inflammatory index, even after the removal of diseased tissue. As a result, analysis of the change in the EHI between the postoperative and later analysis was not helpful. Its greatest value therefore lies in its use at 6 months postoperatively, allowing discrimination between recurrence and maintenance of remission. The EHI was higher in patients with recurrence than those in remission at 6 months. EHI would therefore appear to be a good early marker of recurrence, with accuracy approaching that of faecal calprotectin.

EHI did not discriminate as well at 18 months postoperatively. However, at 18 months the EHI differed significantly and discriminated between the clinically important macroscopic normality [i0], those with any macroscopic disease [≥i1], and those with severe endoscopic recurrence [≥i3]. The Rutgeerts scores of i2a five or more aphthous lesions or larger lesions confined to the ileocolonic anastomosis] or i2b [five or more aphthous lesions or larger lesions in the neoterminal ileum with normal intervening mucosa] were not assessed in this study, due to the low number of patients with i2a disease at 6 [five patients] and 18 months [four patients].^38,39^

Using the EHI threshold [<20] previously reported, at 6 months postoperatively the EHI had an acceptable sensitivity and negative predictive value which is concordant with previous data from general [non-surgical] Crohn’s disease cohorts.^20^ The upper threshold of EHI ≥50 had a high specificity, indicating that nearly all patients with this EHI value did indeed have recurrent endoscopic disease. Such a result would therefore indicate the need for colonoscopic confirmation. The overall AUROC at 6 months for the EHI was comparable to that of calprotectin, indicating the utility of EHI as an early marker of postoperative recurrence in Crohn’s disease.

At 18 months, there was a lower sensitivity for the <20 threshold compared with 6 months, but the upper threshold of ≥50 showed a similar specificity to the 6-month value. The AUROC at 18 months for EHI was 0.52, with calprotectin being superior at this time point. The reasons for this difference in test performance between time points is unclear but may relate to specific markers within the EHI, such as those of matrix remodelling, being most relevant early in the postoperative period. In addition, the difference between EHI sensitivity at 6 and 18 months may relate to unknown confounders or to an increased rate of patient withdrawal between 6 and 18 months limiting sample size. There was also significant treatment heterogeneity in this cohort at later time points, mainly due to the active arm intensifying therapy after the 6-month endoscopic assessment.

At both time points, an EHI <10 ruled out any recurrent disease with a high negative predictive value [83.3–100%] which is comparable to faecal calprotectin [100 µg/g, NPV of 90–93%] in this cohort.^12^ Approximately 10% of patients in this cohort had a EHI of <10 across all time points. The very low false-negative rate means that such an EHI is reassuring that the patient does not have endoscopic recurrence.

We tested three thresholds for recurrence in this study, the standard Rutgeerts cut-off of <i2 vs ≥i2, i0 [mucosal normality] vs i3-4 [severe recurrence], and remission vs severe recurrence [i0-i1 vs i3-i4]. This tested the ability of the EHI to discern mild recurrence [lower disease burden] that may indicate progression, as well as the identification of already severe disease recurrence [higher disease burden]. Whereas recurrence can be ruled in at 6 months with an EHI ≥50, and an EHI <20 is reassuring for the absence of disease, patients who fall within this range should undergo further assessment for disease progression, with a faecal calprotectin or ileocolonoscopy. An EHI threshold of <10 is very sensitive for endoscopic recurrence and may allow avoidance of colonoscopy with a high level of confidence. These test thresholds therefore serve as clinically useful measures for excluding endoscopic disease recurrence, and serial EHI measurements may be helpful for prospectively monitoring changes in disease activity over time.

There is overall concordance between the EHI and the Rutgeerts score when all samples and time points are considered in a linear mixed model. Although the Rutgeerts score is unvalidated, it remains the best predictor of postoperative disease course. An additional advantage to being a minimally invasive test, the EHI may be elevated in the absence of disease seen on ileocolonoscopy, such as more proximal ileal disease. It therefore cannot be ruled out that a proportion of EHI false-positives reflect true proximal small bowel disease recurrence.

Limitations of this analysis include the smaller number of patients who had severe recurrent disease and colonic disease.

EHI can be used to monitor for endoscopic disease status in the postoperative setting despite low volume disease, with results similar to faecal calprotectin. The only other widely available serum marker, CRP, is not able to discern remission versus recurrence in any comparisons. The EHI demonstrates good utility to rule out postoperative recurrence, with a high sensitivity at a threshold of <20 and excellent test performance at a threshold of <10. Whereas use of the EHI is unlikely to replace postoperative endoscopic surveillance completely, it may enable a reduction in colonoscopy frequency in some patients. When used with ileocolonoscopy, with or without faecal calprotectin, a non-invasive multi-modal approach could be used to monitor or disease recurrence. Such a model needs to be tested. For patients with the highest risk of recurrent disease, and therefore the greatest need for colonoscopy, it may have great value. In patients who decline ileocolonoscopy, the EHI test combined with faecal calprotectin measurement will provide early identification of patients with postoperative mucosal disease recurrence. This test adds to the diagnostic armamentarium after Crohn’s disease surgery.

## Supplementary Material

jjac076_suppl_Supplementary_Figure_S1

jjac076_suppl_Supplementary_Figure_S2

jjac076_suppl_Supplementary_Figure_S3A

jjac076_suppl_Supplementary_Figure_S3B

jjac076_suppl_Supplementary_Material

## Data Availability

The de-identified data underlying this article will be shared on reasonable request to the corresponding author. Review by both the requester’s and the authors’ institutional Human Research Ethics Committee may be required.
