# Supplementary figures and images for: Non-invasive Serological Monitoring for Crohn’s Disease Postoperative Recurrence
Source: J Crohns Colitis. 2022 Jun 11;16(12):1797–807. doi: 10.1093/ecco-jcc/jjac076 (PMC9721459; doi:10.1093/ecco-jcc/jjac076)

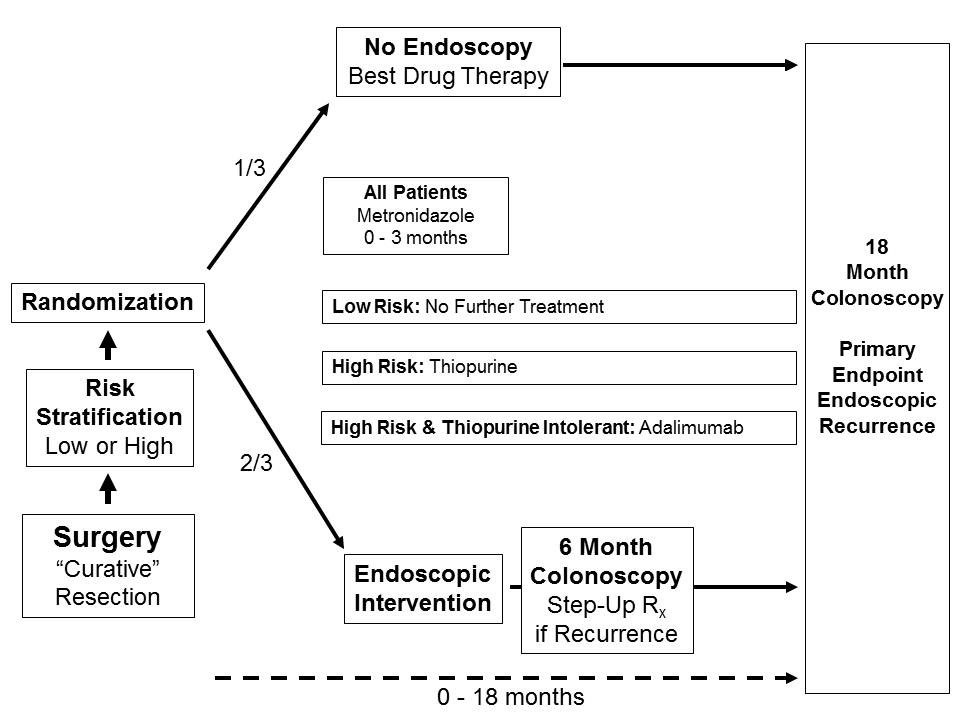

Supplement: jjac076_suppl_Supplementary_Figure_S1 [file jjac076_suppl_supplementary_figure_s1.jpeg]

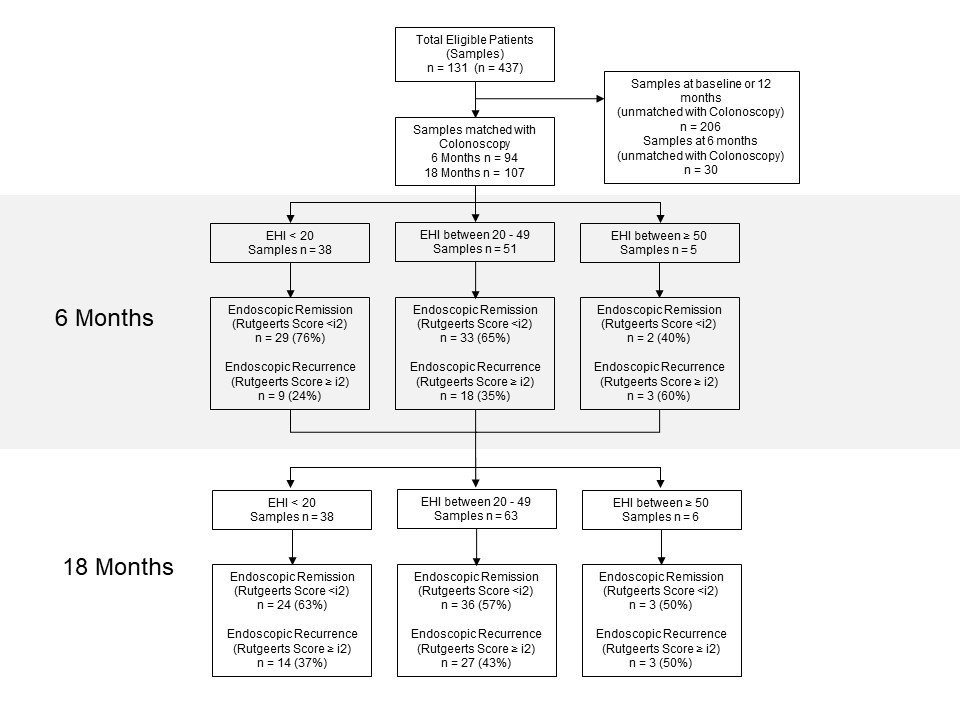

Supplement: jjac076_suppl_Supplementary_Figure_S2 [file jjac076_suppl_supplementary_figure_s2.jpeg]

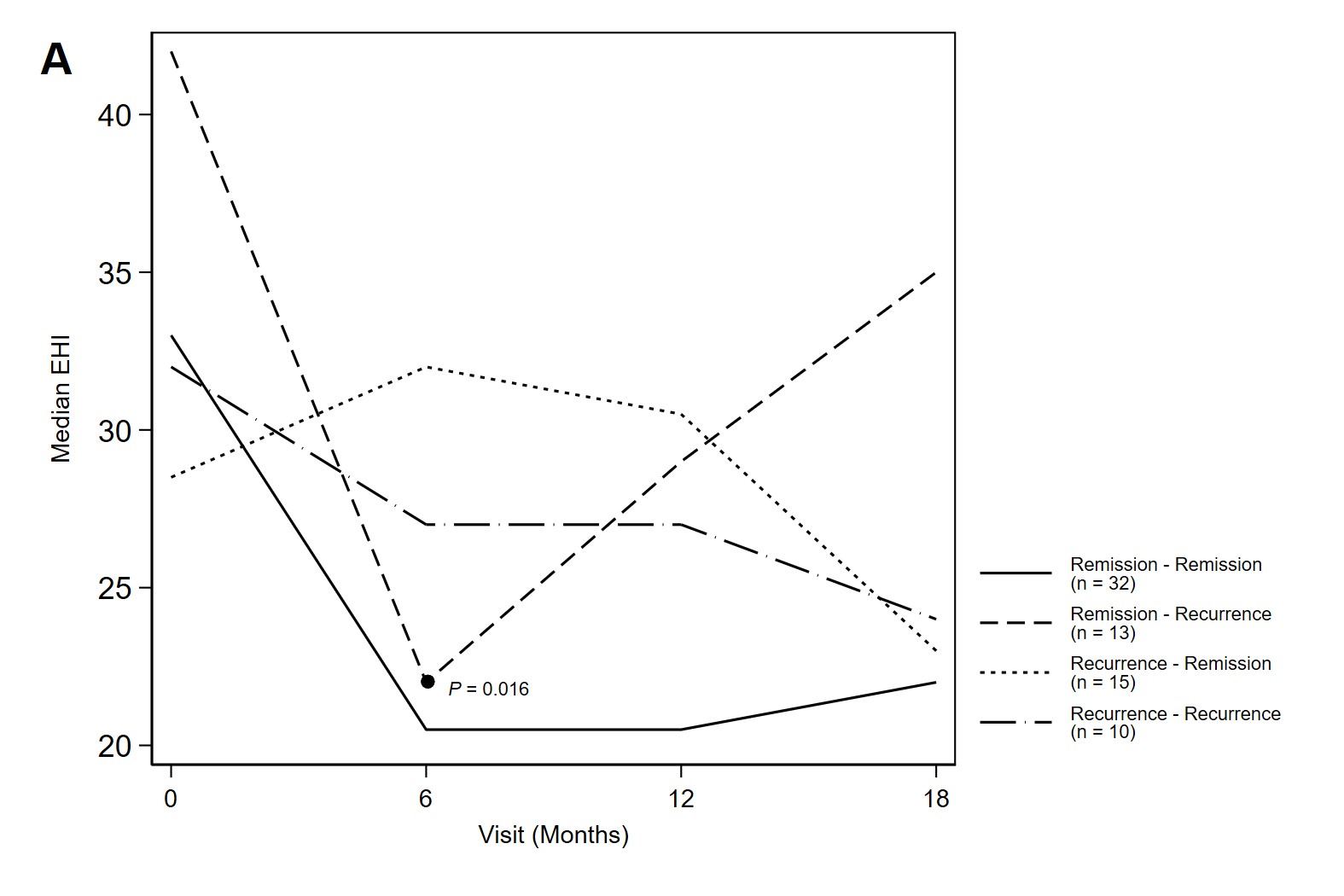

Supplement: jjac076_suppl_Supplementary_Figure_S3A [file jjac076_suppl_supplementary_figure_s3a.jpeg]

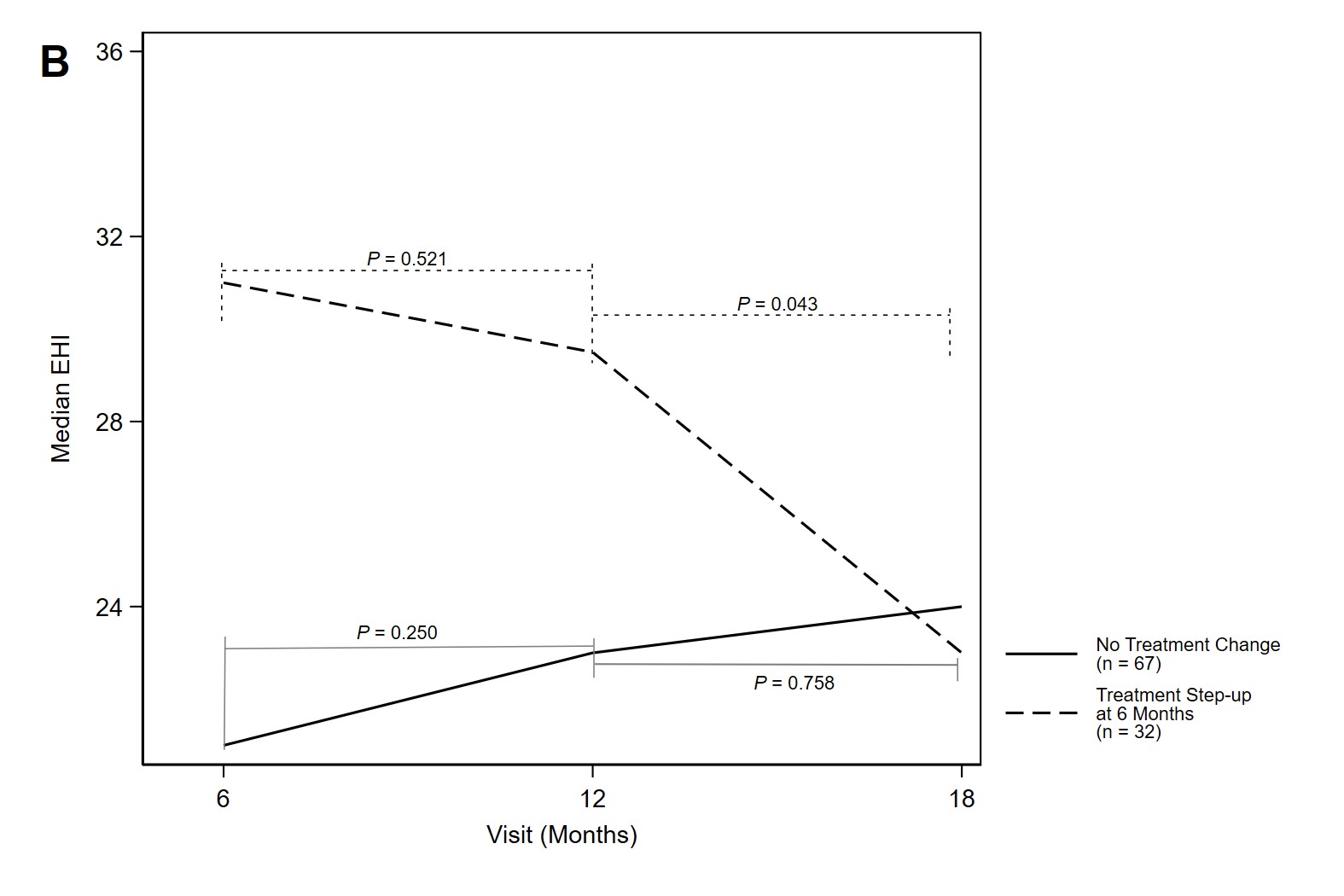

Supplement: jjac076_suppl_Supplementary_Figure_S3B [file jjac076_suppl_supplementary_figure_s3b.jpeg]
