# Supplementary material for: Non-invasive Serological Monitoring for Crohn’s Disease Postoperative Recurrence
Source: J Crohns Colitis. 2022 Jun 11;16(12):1797–807. doi: 10.1093/ecco-jcc/jjac076 (PMC9721459; doi:10.1093/ecco-jcc/jjac076)
Supplement: jjac076_suppl_Supplementary_Material [file jjac076_suppl_supplementary_material.docx]

**Non-Invasive Serological Monitoring for Crohn’s Disease Post-Operative Recurrence**

**SUPPLEMENTARY DATA**

***Predictive value of EHI***

For those in the active arm, the EHI at preoperative baseline did not differ significantly between those with remission or recurrence subsequently at 6 months: i0 or i1 39 [IQR 20-53] vs ≥i2 32 [22-62], n = 48; P = 0.94.

For the cross-sectional POCER study cohort, the EHI at preoperative baseline did not differ significantly between those with remission or recurrence subsequently at 18 months: i0 or i1 29.5 [IQR 15-57.5] vs ≥i2 33 [IQR 24–56], n = 51; P = 0.46.

***Change of EHI over time in relation to endoscopic remission or recurrence***

The dynamics of the EHI over time were assessed in the 70 patients in the active treatment POCER study arm who had a colonoscopy at 6 and 18 months. The patients were divided into four groups based on endoscopic remission or recurrence at 6 and 18 months. Patients who were in endoscopic remission at 6 months and remained in endoscopic remission at 18 months did not show a significant change in the EHI across timepoints. Conversely, patients in endoscopic remission at 6 months who went on to develop endoscopic recurrence at 18 months showed a significant decrease in median EHI in the first 6 months (baseline 42 vs 6 months 22; P = 0.016, supplementary table 2), and a subsequent rise in EHI from 6 to 18 months (6 months 22 vs 18 months 35; P = 0.039, supplementary figure 3, panel A). The EHI did not change significantly in those who had disease recurrence across both 6 and 18 month endoscopies.

EHI significantly decreased between 12 and 18 months in those with recurrence who responded to treatment intensification at 6 months and were in endoscopic remission at 18 months (P = 0.043, supplementary figure 3, panel B).

**Supplementary Table 1. Biomarkers included in Monitr™**

| **Biomarker** | **Abbreviation** | **Pathway** |
| --- | --- | --- |
| Carcinoembryonic antigen-related Cell Adhesion Molecule 1 | CEACAM1 | Cell Adhesion |
| Vascular Cell Adhesion Molecule 1 | VCAM1 | Cell Adhesion |
| C-Reactive Protein | CRP | Inflammation/Acute Phase Reactants |
| Serum Amyloid A1 | SAA1 | Inflammation/Acute Phase Reactants |
| Angiopoietin – 1 | Ang-1 | Angiogenesis |
| Angiopoietin – 2 | Ang-2 | Angiogenesis |
| Matrix Metalloproteinase -1 | MMP-1 | Matrix Remodelling |
| Matrix Metalloproteinase -2 | MMP-2 | Matrix Remodelling |
| Matrix Metalloproteinase -3 | MMP-3 | Matrix Remodelling |
| Matrix Metalloproteinase -9 | MMP-9 | Matrix Remodelling |
| Extracellular Matrix Metalloproteinase Inducer | EMMPRIN | Matrix Remodelling |
| Transforming Growth Factor alpha | TGFα | Cell Proliferation and Repair |
| Interleukin-7 | IL-7 | Immune Recruitment |

**Supplementary Table 2.**

Longitudinal EHI values in 70 active-care study treatment arm patients with both 6 and 18 month colonoscopic outcomes. P values shown for Wilcoxon paired sign-rank test.

| **Status at 6 Months** | **Status at 18 Months** | **N** |  | **Median EHI** | **IQR** | **Δ MHI compared to 6 Months** | **P Value Baseline to 6 Months** | **P Value 6 to 18 Months** |
| --- | --- | --- | --- | --- | --- | --- | --- | --- |
| Remission (≤i1) | Remission (≤i1) | 32 | Baseline (n = 13) | 33 | 15-43 |  | 0.087 |  |
|  |  |  | 6 Months | 20.5 | 12.5-27.3 |  |  | 0.896 |
|  |  |  | 18 Months | 22 | 9.5-29 | +1.5 |  |  |
| Remission (≤i1) | Recurrence (≥i2) | 13 | Baseline (n = 7) | 42 | 27-62 |  | **0.016** |  |
|  |  |  | 6 Months | 22 | 16-33 |  |  | **0.039** |
|  |  |  | 18 Months | 35 | 22-45 | +13 |  |  |
| Recurrence (≥i2) | Remission (≤i1) | 15 | Baseline (n = 8) | 28.5 | 17.25-51.5 |  | 0.483 |  |
|  |  |  | 6 Months | 32 | 20.5-34 |  |  | 0.256 |
|  |  |  | 18 Months | 23 | 18-32.5 | -9 |  |  |
| Recurrence (≥i2) | Recurrence (≥i2) | 10 | Baseline (n = 7) | 32 | 25.5-54 |  | 0.128 |  |
|  |  |  | 6 Months | 27 | 18.75-32 |  |  | 0.285 |
|  |  |  | 18 Months | 24 | 16-27 | -3 |  |  |

**SUPPLEMENTARY FIGURE LEGENDS**

**Supplementary Figure 1.** POCER Study Design [^1^](#_ENREF_1)

**Supplementary Figure 2.** STARD Diagram

**Supplementary Figure 3.**

**Panel A.** EHI over time by response group (active care patients). P values from paired Wilcoxon signed rank test (longitudinal cohort, n = 70).

**Panel B.** Effect of treatment step-up (active care patients, n = 99) vs no treatment change at 6 months on the EHI at subsequent timepoints. P values from paired Wilcoxon signed rank test.

**REFERENCES**

1. De Cruz P, Kamm MA, Hamilton AL, et al. Crohn's Disease Management after Intestinal Resection: A Randomised Trial. Lancet 2015;385:1406-17.
